# Short-chain fatty acids and insulin sensitivity: a systematic review and meta-analysis

**DOI:** 10.1093/nutrit/nuad042

**Published:** 2023-06-08

**Authors:** Nhan H T Pham, Mugdha V Joglekar, Wilson K M Wong, Najah T Nassif, Ann M Simpson, Anandwardhan A Hardikar

**Affiliations:** are with the Diabetes and Islet Biology Group, School of Medicine, Western Sydney University, Campbelltown, New South Wales, Australia; are with the School of Life Sciences, University of Technology Sydney, Ultimo, New South Wales, Australia; are with the Diabetes and Islet Biology Group, School of Medicine, Western Sydney University, Campbelltown, New South Wales, Australia; are with the Diabetes and Islet Biology Group, School of Medicine, Western Sydney University, Campbelltown, New South Wales, Australia; are with the School of Life Sciences, University of Technology Sydney, Ultimo, New South Wales, Australia; are with the School of Life Sciences, University of Technology Sydney, Ultimo, New South Wales, Australia; are with the Diabetes and Islet Biology Group, School of Medicine, Western Sydney University, Campbelltown, New South Wales, Australia; is with the Department of Science and Environment, Roskilde University, Roskilde, Denmark

**Keywords:** acetate, butyrate, HOMA-IR, insulin sensitivity, propionate, short-chain fatty acids, type 2 diabetes

## Abstract

**Context:**

There is substantial evidence that reduced short-chain fatty acids (SCFAs) in the gut are associated with obesity and type 2 diabetes, although findings from clinical interventions that can increase SCFAs are inconsistent.

**Objective:**

This systematic review and meta-analysis aimed to assess the effect of SCFA interventions on fasting glucose, fasting insulin, and homeostatic model assessment of insulin resistance (HOMA-IR).

**Data Sources:**

Relevant articles published up to July 28, 2022, were extracted from PubMed and Embase using the MeSH (Medical Subject Headings) terms of the defined keywords [(short-chain fatty acids) AND (obesity OR diabetes OR insulin sensitivity)] and their synonyms. Data analyses were performed independently by two researchers who used the Cochrane meta-analysis checklist and the PRISMA guidelines.

**Data Extraction:**

Clinical studies and trials that measured SCFAs and reported glucose homeostasis parameters were included in the analysis. Standardized mean differences (SMDs) with 95%CIs were calculated using a random-effects model in the data extraction tool Review Manager version 5.4 (RevMan 5.4). The risk-of-bias assessment was performed following the Cochrane checklist for randomized and crossover studies.

**Data Analysis:**

In total, 6040 nonduplicate studies were identified, 23 of which met the defined criteria, reported fasting insulin, fasting glucose, or HOMA-IR values, and reported change in SCFA concentrations post intervention. Meta-analyses of these studies indicated that fasting insulin concentrations were significantly reduced (overall effect: SMD = −0.15; 95%CI = −0.29 to −0.01, P = 0.04) in treatment groups, relative to placebo groups, at the end of the intervention. Studies with a confirmed increase in SCFAs at the end of intervention also had a significant effect on lowering fasting insulin (P = 0.008). Elevated levels of SCFAs, compared with baseline levels, were associated with beneficial effects on HOMA-IR (P < 0.00001). There was no significant change in fasting glucose concentrations.

**Conclusion:**

Increased postintervention levels of SCFAs are associated with lower fasting insulin concentrations, offering a beneficial effect on insulin sensitivity.

**Systematic Review Registration:**

PROSPERO registration number CRD42021257248.

## INTRODUCTION

Type 2 diabetes mellitus (T2DM) is characterized by a reduction in β-cell function as well as by insulin resistance, wherein skeletal muscle, liver, and adipocytes cannot uptake sufficient amounts of glucose.^1^ It leads to life-threatening complications such as neuropathy, retinopathy, and cardiovascular disease. According to the World Health Organization, there are over 537 million adults living with T2DM, and this number is estimated to reach 783 million by 2045.^2^

Along with T2DM, overweight (body mass index [BMI] of 25 to < 30 kg/m^2^) and obesity (BMI ≥ 30) are considered major health problems, currently affecting approximately 2 billion people worldwide.^3^^,^^4^ Obesity is a major risk factor for the development of cardiovascular disease, hypertension, and stroke. It is also known to dramatically increase the risk of T2DM, as 90% of individuals with T2DM are obese or overweight.^5^ Beneficial effects of gut microbiota and short-chain fatty acids (SCFAs) on metabolic health in obesity^6^^,^^7^ and T2DM^8^^,^^9^ have been reported. Differences of the diversity of the gut microbiome are well recognized between lean and obese individuals^6^^,^^7^ as well as between individuals with and without diabetes.^10^^,^^11^ Gut microbes produce SCFAs, mainly acetate, propionate, and butyrate, through the fermentation of resistant starch or soluble fiber. These molecules induce the expression of gut hormones such as peptide tyrosine-tyrosine (PYY) and glucagon-like peptide 1 (GLP-1) via free fatty acid receptors (G-protein–coupled receptors 41 and 43),^12^^,^^13^ leading to appetite suppression^14^ and improvement of glucose tolerance and insulin sensitivity.^15^^,^^16^ In addition, fecal transplantation from lean donors to obese recipients altered the gut microbial composition and improved insulin sensitivity in the recipients.^17^^,^^18^

Short-chain fatty acids can be administered directly, as sodium salts or esters, or indirectly, as pre-/probiotics or high-fiber diets. Multiple clinical trials have aimed at increasing SCFA levels in participants with impaired insulin sensitivity, preexisting overweight/obesity, or multiple gastrointestinal abnormalities. Many meta-analyses of SCFAs in human clinical trials are available, but most of these focus on irritable bowel disease,^19–21^ inflammatory markers,^22^^,^^23^ obesity,^24^^,^^25^ or gut biota.^26^^,^^27^ Relevant previous meta-analyses (see Table S1 in the Supporting Information online) report effects on insulin and glucose concentrations after consumption of specific diets such as those high in fiber, resistant starch or whole grains, or synbiotics and probiotics. Only one review analyzed changes in total SCFAs, along with levels of *Bifidobacteria* and fasting glucose; however, it did not examine the effect of SCFA changes on fasting insulin or homeostatic model assessment of insulin resistance (HOMA-IR). Until now, there is no systematic review and meta-analysis that assessed the effects of postintervention changes in SCFAs on insulin sensitivity in humans. To address this research gap, clinical studies (randomized clinical trials, observational studies, or treatment-only studies) that reported actual values of glucose, insulin, or HOMA-IR and that measured SCFAs following interventions were analyzed. This systematic review and meta-analysis includes different interventions that have the potential to alter SCFAs and is focused on studies that confirm a change in SCFA concentrations post intervention.

## METHODS

### Literature search

**Figure 1:**
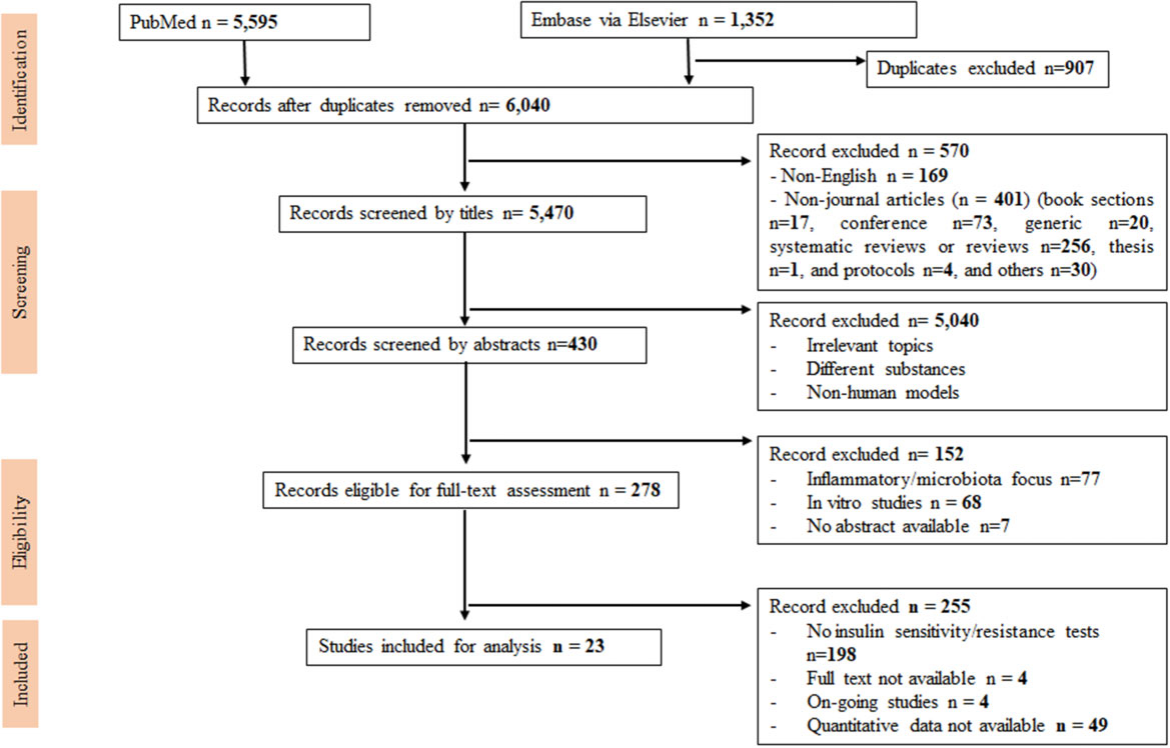
Flow diagram of the literature search process.

A systematic search was carried out using PubMed and Embase via Ovid for studies published from January 1, 1959, to July 28, 2022. There were no restrictions on the use of MeSH (Medical Subject Heading) terms and their synonyms. The keywords used for the literature search were “short-chain fatty acids” AND (“obesity” OR “diabetes” OR “insulin sensitivity”). All studies were screened by title, abstract, and full-text reading (Figure 1), following the Cochrane meta-analysis checklist and the PRISMA (Preferred Reporting Items for Systematic reviews and Meta-Analyses) guidelines^28^ (see Appendix S1 in the Supporting Information online). Descriptions of search strategy, study eligibility, data extraction, and analysis of this systematic review were preregistered in the PROSPERO system (ID no. CRD42021257248).

### Inclusion and exclusion criteria

Papers were included for screening if they contained the keywords (as defined in the *Literature search* section above) and met the PICOS (Participants, Intervention, Comparator, Outcomes, Study design) criteria summarized in Table 1. The following exclusion criteria were applied: (1) studies other than human studies, such as animal studies or cell culture studies, (2) studies not published in the English language, and (3) publication types other than peer-reviewed original papers, such as systematic reviews, meta-analyses, reviews, conference publications, non-article papers, generic writing, theses or chapters, and case reports, (4) studies not providing data for fasting glucose, fasting insulin, or HOMA-IR, and (5) studies not reporting pre- and postintervention SCFA changes.

**Table 1:** PICOS criteria for inclusion and exclusion of studies

Parameter	Inclusion criteria	Exclusion criteria
Participants	Healthy adults, obese/overweight adults, or adults with underlying conditions	Animal and in vitro cellular studies
Interventions	Direct SCFAs interventions (oral/IV infusion/enema of sodium acetate, propionate, or butyrate, alone or mixed) or dietary supplementation (resistant starch, inulin, or chemically modified fiber)	Direct supplementation with substances other than the 3 SCFAs (acetate, propionate, or butyrate)
Comparators	Placebo vs intervention (endpoint); baseline vs endpoint (intervention group)	Two different treatments or doses or intermediate time points
Outcomes	Concentrations of fasting insulin, fasting glucose, or HOMA-IR as well as reporting of changes in plasma or fecal levels of SCFAs (concentrations shown as bar graphs or in text) at end of intervention	Studies not reporting concentrations of fasting insulin, fasting glucose, or HOMA-IR (eg, AUCs, regression/correlation coefficients, bar graphs, difference in concentrations) and studies not reporting the change in SCFAs
Study design	Randomized clinical trials, crossover studies, or treatment-only studies	Non-original papers (reviews, systematic reviews, conference reports, clinical trial protocols, ongoing studies) and non-English papers

*Abbreviations:* AUC, area under the curve; SCFAs, short-chain fatty acids; HOMA-IR, homeostatic model assessment of insulin resistance; IV, intravenous.

### Data extraction

Results of the literature search were added to an EndNote X8^29^ library and entered into an MS Excel worksheet for data screening and extraction (Figure 1). The first step of removing duplicate hits from the two databases (Embase and PubMed) was performed in the EndNote library, where papers with identical titles and authors were marked as duplicates and removed. In the screening step, titles and abstracts were assessed for eligibility, and articles were excluded if they met any of the exclusion criteria. The full-text versions of the 23 qualifying papers that met the inclusion criteria (original clinical studies in humans; provided values, not area under the curve [AUC] values or coefficients) of fasting glucose or fasting insulin or the HOMA-IR score; and reported change in plasma or fecal SCFAs before and after the intervention were then reviewed and included in the meta-analysis. All values were converted to mean ± standard deviation (SD) for glucose (mmol/L) and insulin (μU/mL). Reported units for glucose and insulin were made consistent.^30^ In 2 of the 23 studies,^31^^,^^32^ units or standard deviation values were confirmed with the authors of the original articles via E-mail correspondence. Subgroup analyses were performed to investigate differences in fasting insulin, glucose, or HOMA-IR between placebo and treatment groups, or between the baseline and the endpoint. Two researchers (N.H.T.P. and M.V.J.) performed the search and data extraction independently and then cross-checked the results.

### Quality of evidence and risk of bias

Risk of bias and the quality of evidence were assessed in accordance with the Cochrane recommendations,^33^ and outcomes were presented using RevMan software, version 5.4.^34^ The criteria included random sequence generation (selection bias), allocation concealment (selection bias), blinding of participants and personnel (performance bias), blinding of outcome assessment (detection bias), incomplete outcome data (attrition bias), selective bias (reporting bias), and other bias. Each criterion was graded as having high, low, or unclear risk of bias. Treatment-only studies were not evaluated for selection, performance, or detection bias because all participants and associated clinicians were aware of the treatment allocation. Several of the studies had a crossover design. Therefore, 3 additional criteria for crossover studies were assessed: appropriate crossover design, carryover effects, and unbiased data (reporting of data at all stages of the trial).^35^

### Data synthesis and statistical analysis

Data synthesis and statistical analysis were carried out using Microsoft Excel and RevMan 5.4 software. Microsoft Excel was used to tabulate and summarize the insulin and glucose values at appropriate units. RevMan 5.4 was used to produce forest plots. All data were entered as mean ± SD. The random-effects model and the inverse variance statistical method were used to calculate standardized mean differences (SMDs) with 95%CIs between the placebo group and the intervention group, or between the baseline and the endpoint. The same analysis was also carried out using the fixed-effects model to ensure the robustness of the calculations. All the analyses were performed in RevMan 5.4. A *P* value < 0.05 was considered significant. Standardized mean difference values for SCFAs, insulin, and glucose were calculated in RevMan 5.4 and used for Spearman correlation analysis. A correlation matrix was generated using the corrplot (version 0.92), Hmisc (version 4.6-0), and dplyr (version 1.0.7) packages and R (version 3.6.1) in Rstudio (version 2021.09.0, Build 351; R Foundation for Statistical Computing, Vienna, Austria). The correlation significance was analyzed and verified on GraphPad Prism version 9.4.1 (GraphPad Software; San Diego, CA, USA).

## RESULTS

### Characteristics of studies included in meta-analysis

Figure 1 shows the PRISMA flow diagram for the stepwise selection of studies. The initial search using the defined MeSH terms in PubMed and Embase via Ovid identified 6040 papers (after excluding 907 duplicates). During screening, 570 papers were removed because they were not published in English or were non-journal articles. After the remaining 5470 papers were screened by title and abstract, 278 were deemed eligible for full-text assessment. A majority of these studies (n = 198) were excluded because insulin sensitivity/resistance tests were not performed, while another 49 did not present quantitative data on fasting insulin or fasting glucose. The 23 studies that contained actual values for fasting glucose and insulin were included in the meta-analysis (Table 2^31^^,^^32^^,^^36–56^).

**Table 2:** Details of the 23 studies included in the meta-analysis

Study design	Reference; country	Intervention; route; dosage; duration	Participants in intervention group^a^				Participants in placebo group^a^				Postintervention SCFA levels (source of SCFAs; method of measurement)	Effects of intervention on glucose/insulin parameters
			Participants	Sex	Age (years)	BMI (kg/m^2^) or body mass (kg)	Participants	Sex	Age (years)	BMI (kg/m^2^) or body mass (kg)		
Treatment only	Burnier et al (1992)^31^; Switzerland	Sodium acetate; IV infusion; 0.5 M acetate (at rate of 2.5 mmol/min); 120 min	9 healthy individuals	6 M and 3 F	30 ± 1	70 ± 3 (kg)	–	–	–	–	↑ Acetate (plasma; acetate kinase method)	Plasma insulin and glucose were not changed during acetate infusion.
	Fava et al (2013)^42^; UK	High-saturated-fat diet; diet; high-saturated-fat diet containing 38%E from fat; 24 wk	11 individuals with metabolic syndrome	N/A	54 ± 9.5	28.8 ± 4.9 (kg/m^2^)	–	–	–	–	All SCFAs ↑ (feces; GC-MS chromatography, FFAP column)	Fasting plasma glucose and insulin were not significantly altered after high-saturated-fat diet.
	Härma et al (2021)^45^; Finland	Gastric bypass (RYGB); surgery; –; 6 mo	30 obese (T2DM and nondiabetic) individuals	9 M and 21 F	47.3 ± 8.8	44.5 ± 5.7 (kg/m^2^)	–	–	–	–	↓ Acetate and ↓ butyrate, no change in propionate (feces; GC-MS)	All individuals undergoing surgery had improved glycemic control.
	González Hernández et al (2020)^44^; Netherlands	Low-calorie diet; diet; low-calorie diet with 800 kcal/d; 8 wk	478 individuals with BMI ≥ 27	175 M and 303 F	41 ± 6	35 ± 5 (kg/m^2^)	–	–	–	–	↑ Acetate (plasma; 1H NMR spectroscopy)	In females, a positive association was observed between changes in acetate and changes in HOMA-IR.
	Akamine et al (2022)^32^; Japan	White rice amazake (WA) or brown rice amazake (BA); diet; 350 g/d; 4 wk	19 individuals with metabolic syndrome (WA); 21 individuals with metabolic syndrome (BA)	11 M and 8 F (WA); 9 M and 12 F (BA)	56.7 ± 2.2 (WA); 58.5 ± 2.2 (BA)	28.8 ± 0.8 (WA); 28.8 ± 0.7 (BA)	–	–	–	–	No change in any SCFAs (plasma; GC-MS chromatography)	Plasma glucose and plasma insulin were not significantly different between the 2 treatments.
Randomized, placebo-controlled, parallel-arm studies	Chambers et al (2015)^41^; UK	Inulin propionate ester; oral; inulin, 10 g/d (control) vs inulin-propionate ester, 10g/d (intervention); 24 wk	25 overweight individuals	10 M and 15 F	55.3 ± 1.4	88.5 ± 2.9 (kg)	24 overweight individuals	9 M and 15 F	53.4 ± 1.5	91.0 ± 2.8 kg	↑ Acetate, ↑ propionate, no change in butyrate (feces; GC-FID chromatography)	Fasting glucose, fasting insulin, and postprandial responses were not significantly different post intervention.
	Vetrani et al (2016)^54^; Italy	Whole-grain diet; diet; refined cereal products (control) vs whole-grain cereal (intervention); 12 wk	21 healthy individuals	9 M and 12 F	57.2 ± 1.9	32.1 ± 1.4 (kg/m^2^)	19 healthy individuals	7 M and 12 F	58.4 ± 1.6	31.5 ± 1.3 kg/m^2^	No change in acetate or butyrate, ↑ propionate (plasma; GC-MS chromatography)	Whole-grain wheat-based diet lowered postprandial insulin concentrations.
	Canfora et al (2017)^39^; Netherlands	GOS; diet; maltodextrin, 16.95 g/d (control) vs GOS 15 g/d (intervention); 12 wk	21 obese or overweight individuals	11 M and 10 F	59.2 ± 7.2	98.4 ± 11.9 (kg)	23 obese or overweight individuals	12 M and 11 F	58.4 ± 7.3	96.9 ± 11.5 kg	No change in any SCFAs (plasma and feces; GC-MS chromatography)	Intervention with GOS did not alter insulin sensitivity.
	Chambers et al (2019(2))^56^; UK	Inulin propionate ester; oral; inulin, 20 g/d (control) vs inulin-propionate ester (intervention), 20 g/d; 6 wk	9 individuals with NAFLD	4 M and 5 F	51 ± 4	31.5 ± 1.9 (kg/m^2^)	9 individuals with NAFLD	5 M and 4 F	49 ± 4	29.5 ± 1.4 kg/m^2^	No change in acetate or propionate, ↑ butyrate (plasma; 1H NMR spectroscopy)	Change in HOMA-IR was significantly different post intervention.
	Palacios et al (2020)^51^; Australia	Probiotic; oral; 200 mg microcrystalline cellulose + 10 mg silica + 10 mg magnesium stearate (control) vs multistrain probiotic (intervention); 12 wk	30 individuals with prediabetes or T2DM	17 M and 13 F	61.4 ± 8.9	35.5 ± 6.2 (kg/m^2^)	30 individuals with prediabetes or T2DM	11 M and 19 F	56.1 ± 12.3	36.3 ± 7.5 kg/m^2^	No change in any SCFAs (plasma; GC-MS chromatography)	No significant differences in fasting plasma glucose (placebo vs intervention).
	Meslier et al (2020)^49^; Italy	Mediterranean diet; diet; habitual diet (control) vs an individually tailored diet (intervention); 8 wk	43 healthy overweight individuals	21 M and 22 F	43 ± 13	30.9 ± 3.8 (kg/m^2^)	39 healthy overweight individuals	18 M and 21 F	42 ± 12	31.2 ± 5.3 kg/m^2^	No change in any SCFAs (feces; GC-MS chromatography)	No changes observed in blood glucose or plasma insulin concentrations.
	Oh et al (2021)^55^; Korea	Probiotic; oral; 1 capsule daily of microcrystalline cellulose (control) vs 1 capsule daily of *L plantarum* HA01, 4 × 10^9^ CFU (intervention); 8 wk	20 individuals with impaired glucose tolerance	6 M and 14 F	56.4 ± 11.57	25.25 ± 3.14 (kg/m^2^)	20 individuals with impaired glucose tolerance	3 M and 17 F	53.55 ± 10.18	25.03 ± 1.92 kg/m^2^	No change in any SCFAs (feces; LC-triple Q-MS analysis)	Beneficial effect of single-strain probiotic supplementation administered over 8 wk on HbA_1c_ levels in prediabetic individuals.
	Mocanu et al (2021)^50^; Canada	Fiber; diet; microcrystalline cellulose, 33 g/d (M) and 27 g/d (F) low fermentable fiber (control) vs same dose of corn fiber, resistant wheat starch type 4, and acacia gum (intervention); 6 wk	15 obese individuals	2 M and 13 F	48.4 ± 8.8	131.3 ± 32.0 (kg)	17 obese individuals	5 M and 12 F	48.3 ± 10.4	122.8 ± 25.5 kg	No change in any SCFAs (feces; GC-FID chromatography)	No difference in HOMA-IR observed between baseline and end of intervention in the treatment group.
Randomized, single-blind (Alles et al^36^) or double-blind (all others), placebo-controlled, crossover studies	Alles et al (1999)^36^; Netherlands	FOS; diet; glucose, 4 g/d (control) vs FOS, 15 g/d (intervention); 20 d	20 patients with T2DM	9 M and 11 F	59.3 ± 5.4	28.3 ± 3.5 (kg/m^2^)	20 patients withT2DM	9 M and 11 F	59.3 ± 5.4	28.3 ± 3.5 kg/m^2^	No change in acetate (plasma; enzymatic method)	Dietary supplementation did not change blood glucose in patients with T2DM.
	McIntosh et al (2003)^48^; Australia	High-fiber wheat or rye vs low fiber; diet; dietary fiber, 19 g/d (control) vs 32 g/d (intervention); 4 wk	28 overweight individuals	28 M	40–65	30 ± 0.9 (kg/m^2^)	28 overweight individuals	28 M	40–65	30 ± 0.9 kg/m^2^	Unchanged acetate, ↑ propionate in high-wheat group, ↑ butyrate in high-rye group (feces; GC-MS or LC-MS chromatography)	Improved metabolic health and ↓ plasma insulin in intervention compared with control.
	Sandberg et al (2016)^52^; Sweden	Rye-based diet; diet; 100% white wheat flour bread (control) vs 85% whole rye kernels + 15% white wheat flour bread (intervention); 1 or 3 consecutive meals	19 healthy recruits (data available for 18)	9 M and 10 F	25.6 ± 3.5	21.9 ± 1.87 (kg/m^2^)	19 healthy recruits (data available for 17)	9 M and 10 F	25.6 ± 3.5	21.9 ± 1.87 kg/m^2^	All SCFAs ↑ (plasma; GC chromatography)	Rye-kernel-based meal ↓ glycemia and improved insulin sensitivity.
	Sandberg et al (2018)^53^; Sweden	Rye-kernel and resistant starch type 2; diet; 100% white wheat flour bread (control) vs 43% rye kernels + 43% whole-grain rye flour + 14% HAM-RS2 flour bread (intervention); 3 d	38 healthy individuals	8 M and 30 F	63.9 ± 5.5	24.2 ± 2.5 (kg/m^2^)	38 healthy individuals	8 M and 30 F	63.9 ± 5.5	24.2 ± 2.5 kg/m^2^	All SCFAs ↑ (plasma; GC-MS chromatography)	Whole-grain rye-based bread ↑ insulin sensitivity and fasting SCFAs but not fasting insulin or fasting glucose.
	Johnstone et al (2020)^46^; UK	Resistant starch; diet; digestible starch, 22 g/d for F and 26 g/d for M (control) vs resistant starch type 3, 22 g/d for F and 26 g/d for M; 10 d	19 obese or overweight individuals	11 M and 8 F	20–62	94.3 ± 18.5 (kg)	19 obese or overweight individuals	11 M and 8 F	20–62	94.3 ± 18.5 kg	No change in any SCFAs (feces, capillary GC-MS chromatography)	Fasting blood glucose, but not insulin, was lower in resistant starch diet.
	Ampatzoglou et al (2015)^37^; UK	Whole-grain diet; diet; whole grain, < 16 g/d (control) vs > 80 g/d (intervention); 6 wk	33 healthy individuals	12 M and 21 F	48.8 ± 1.1	27.9 ± 0.7 (kg/m^2^)	33 healthy individuals	12 M and 21 F	48.8 ± 1.1	27.9 ± 0.7 kg/m^2^	No change in any SCFAs (feces; HPLC)	No significant changes in fecal SCFAs or blood glucose following whole grain consumption.
	Giacco et al (2004)^43^; Italy	Short-chain FOS; diet; short-chain FOS, 15 g/d (placebo) vs 10.6 g/d (intervention); 2 mo	30 patients with mild hypercholesterolemia	20 M and 10 F	45.5 ± 9.9	26.6 ± 2.2 (kg/m^2^)	30 patients with mild hypercholesterolemia	20 M and 10 F	45.5 ± 9.9	26.6 ± 2.2 kg/m^2^	No change in acetate (plasma; GC-MS chromatography)	Postprandial insulin response (but not glucose) ↓ significantly post intervention.
	Maki et al (2012)^47^; USA	Resistant starch type 2; diet; HAM-RS2, 0 g/d (control) vs 30 g/d (intervention); 4 wk	33 overweight or obese individuals	11 M and 22 F	49.5 ± 1.6	30.6 ± 0.5 (kg/m^2^)	33 overweight or obese individuals	11 M and 22 F	49.5 ± 1.6	30.6 ± 0.5 kg/m^2^	↑ acetate (in F), no change in propionate or butyrate (plasma; GC chromatography)	Insulin sensitivity improved in males but not in females.
	Chambers et al (2019)^40^; UK	Inulin propionate ester; oral; cellulose, 20 g/d (control) vs inulin-propionate ester, 20 g/d (intervention); 6 wk	12 nondiabetic individuals with obesity or overweight	3 M and 9 F	60 ± 1	29.8 ± 0.9 (kg/m^2^)	12 nondiabetic individuals with obesity or overweight	3 M and 9 F	60 ± 1	29.8 ± 0.9 kg/m^2^	No change in any SCFAs (plasma; 1H NMR spectroscopy)	Colonic propionate delivery via inulin propionate ester improved insulin sensitivity.
	Byrne et al (2019)^38^; UK	Inulin propionate ester; oral; inulin, 10 g/d (control) vs inulin-propionate ester, 10 g/d (intervention); 6 d	21 healthy individuals with obesity or overweight	9 M and 12 F	59 ± 8	81.5 ± 9 (kg)	21 healthy individuals with obesity or overweight	9 M and 12 F	59 ± 8	81.5 ± 9 kg	No change in any SCFAs (plasma; GC-MS chromatography)	No change in baseline values or AUC for glucose or insulin after intervention.

*Abbreviations and symbols*: AUC, area under the curve; CFU, colony-forming units; F, female; FOS, fructo-oligosaccharides; GC, gas chromatography; GC-FID, gas chromatography–flame ionization detection; GC-MS, gas chromatography–mass spectrometry; GOS, galacto-oligosaccharides; 1H NMR, proton nuclear magnetic resonance; HAMRS2, high-amylose maize resistant starch type 2; HbA_1c_, hemoglobin A_1c_; HOMA-IR, homeostatic model assessment of insulin resistance; HPLC, high-performance liquid chromatography; IV, intravenous; LC-MS, liquid chromatography–mass spectrometry; LC-Triple Q-MS, liquid chromatography triple quadrupole mass spectrometry; M, male; NAFLD, nonalcoholic fatty liver disease; RYGB, Roux-en-Y gastric bypass; SCFAs, short-chain fatty acids; T2DM, type 2 diabetes mellitus; ↑, increased; ↓, decreased.

a Reported per the original article.

**Figure 2:**
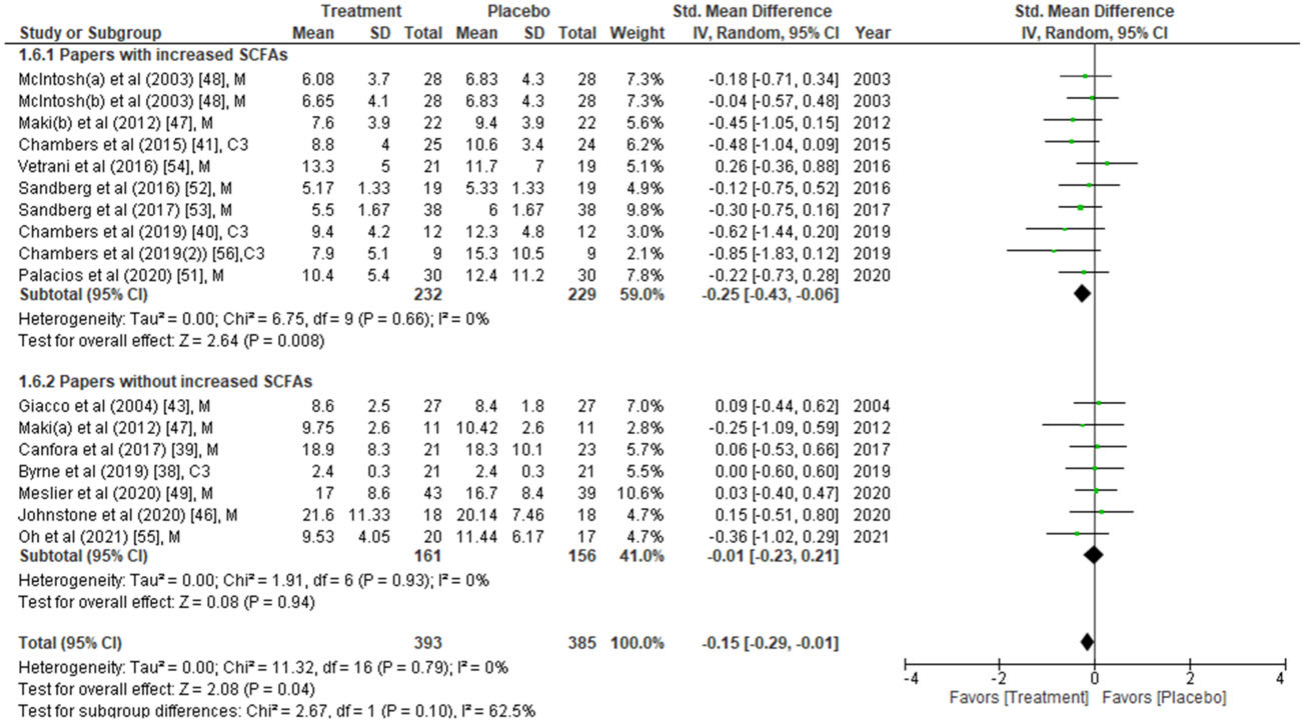
**Fasting insulin concentrations from each study were compared between placebo group and treatment group at the end of the intervention period**. Data are presented as the standardized mean difference (SMD) in fasting insulin (µU/mL) and have been separated into two subgroups: one with evidence of an increase in short-chain fatty acids (SCFAs) concentration(s) post intervention, and the other without any change in SCFAs post intervention. The type of intervention (M, meal/mixed; C3, propionate) is noted at the end of each study. *Abbreviation*: IV, inverse variance.

**Figure 3:**
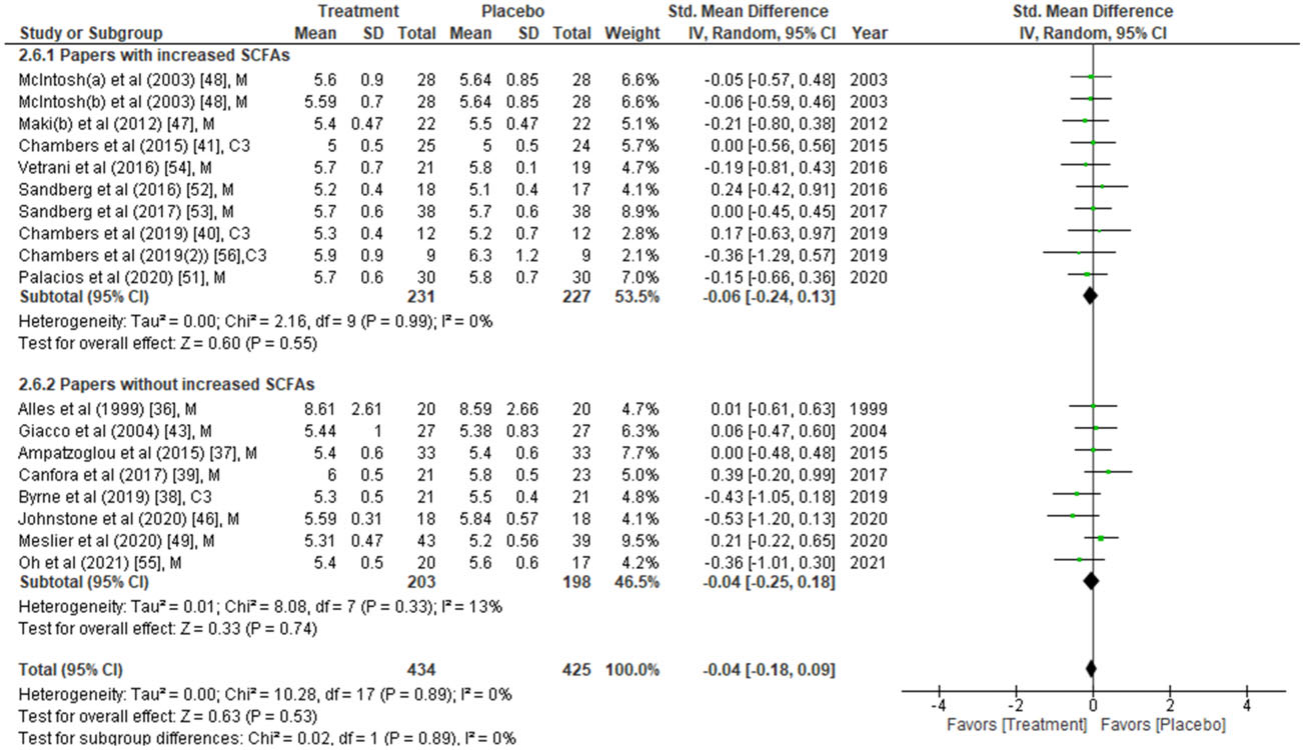
**Fasting glucose concentrations from each study were compared between placebo group vs treatment group at the end of the intervention period.** Data are presented as the standardized mean difference (SMD) in fasting glucose (mmol/L) and have been separated into two subgroups: one with evidence of an increase in short-chain fatty acids (SCFAs) concentration(s) post intervention, and the other without any change in SCFAs post intervention. The type of intervention (M, meal/mixed; C3, propionate) is noted at the end of each study. *Abbreviation:* IV, inverse variance.

**Figure 4:**
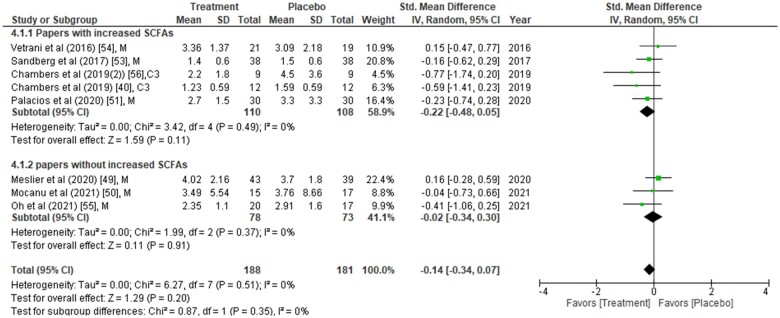
**Homeostatic model assessment of insulin resistance (HOMA-IR) values were compared between placebo group and treatment group at the end of the intervention period.** Data are presented as the standardized mean difference (SMD) in HOMA-IR and have been separated into two subgroups: one with evidence of an increase in short-chain fatty acids (SCFAs) concentration(s) post intervention, and the other without any change in SCFAs post intervention. The type of intervention (M, meal/mixed; C3, propionate) is noted at the end of each study. *Abbreviation*: IV, inverse variance.

Table 2 summarizes the 23 studies included in the meta-analysis. Analyses include data on a total of 1186 study participants from all categories (healthy, 12%; obese/overweight, 67%; and people with underlying conditions, including diabetes, 21%). The type of intervention was dietary in 17 studies, propionate (provided as inulin propionate ester) in 4 studies, gastric bypass in 1 study, and sodium acetate infusion in 1 study. The duration of intervention in most of the studies ranged from 10 days to 6 months but was less than 1 week in 4 studies. Either one or all 3 major SCFAs (acetate, propionate, and butyrate), obtained from plasma or feces, were measured in all studies using chromatography, spectroscopy, or enzymatic methods. Although plasma/fecal SCFAs were measured in all 23 studies, not all studies reported the actual SCFA concentrations. It was therefore difficult to perform a meta-analysis of SCFA concentrations in different interventions. Therefore, the analysis was divided into two subgroups (one with increased SCFAs post intervention and the other without increased SCFAs post intervention;Figure 2,^38–41^^,^^43^^,^^46–49^^,^^51–56^Figure 3,^36–41^^,^^43^^,^^46–49^^,^^51–56^ and Figure 4^40^^,^^49–51^^,^^53–56^).

### Effects of SCFAs on glucose homeostasis parameters between placebo and intervention groups at the endpoint

The effects of SCFAs on fasting insulin (Figure 2), fasting glucose (Figure 3), and HOMA-IR (Figure 4) are stratified for studies (from Table 2) that confirm a significant increase or no change in SCFA concentrations post intervention. In a comparison between placebo and intervention groups at the endpoint (final assessment after intervention), fasting insulin was significantly lower (SMD = −0.25; 95%CI = −0.43 to −0.06; *P* = 0.008) in the treatment group when SCFA concentrations were confirmed to have increased at the end of the intervention (Figure 2). The overall effect on fasting insulin concentrations was also significant (SMD = −0.15; 95%CI = −0.29 to −0.01; *P* = 0.04; Figure 2). In contrast, fasting glucose (SMD = −0.06; 95%CI = −0.24 to 0.13; *P* = 0.55; Figure 3) and HOMA-IR (SMD = −0.22; 95%CI = −0.48 to 0.05; *P* = 0.11; Figure 4) did not differ significantly between treatment and control arms for studies wherein SCFA concentrations increased post intervention. For the subgroup of studies that did not show an increase in SCFAs, there were no significant differences in fasting insulin, fasting glucose, or HOMA-IR between the control and treatment arms (Figures 2, 3, and 4).

When fasting insulin values were analyzed on the basis of direct SCFA administration or indirect dietary treatment, a significant reduction in fasting insulin was observed only after direct intervention (SMD = −0.39; 95%CI = −0.74 to −0.05; *P* = 0.03) but not after the indirect (dietary) intervention compared with their controls (see Figure S1 in the Supporting Information online).^38–41^^,^^43^^,^^46–49^^,^^51–56^ Further subgrouping into direct intervention with evidence of increased SCFAs showed significant reduction of fasting insulin (SMD = −0.58; 95%CI = −1.01 to −0.16; *P* = 0.007) (see Figure S2 in the Supporting Information online).^38–41^^,^^43^^,^^46–49^^,^^51–56^ However, it should be noted that all 3 studies included in this analysis were from the same group of authors and evaluated the inulin-propionate ester intervention.^40^^,^^41^^,^^56^ Similar subanalyses for the source of SCFAs (feces or plasma) revealed that elevated plasma SCFAs were associated with a significant reduction in fasting insulin concentrations (SMD = −0.22; 95%CI = −0.43 to −0.01; *P* = 0.04; see Figure S3 in the Supporting Information online).^38–41^^,^^43^^,^^46–49^^,^^51–56^ Since the beneficial effects on glycemic parameters were seen when SCFAs were higher, a correlation analysis on the changes in SCFA, insulin, and glucose concentrations between placebo and intervention at the endpoint was performed (see Figure S4 in the Supporting Information online). Of the 23 studies, only 8 could be included for this analysis because only those studies reported the actual values of SCFA concentrations (see Table S2 in the Supporting Information online).^36^^,^^37^^,^^40^^,^^43^^,^^47^^,^^48^^,^^52^^,^^56^ Notably, the SMD of acetate between the placebo and intervention groups was significantly and inversely correlated with that of fasting insulin (*r* = −0.64; *P* = 0.048).

### Changes in glucose, insulin, or HOMA-IR relative to baseline

**Figure 5:**
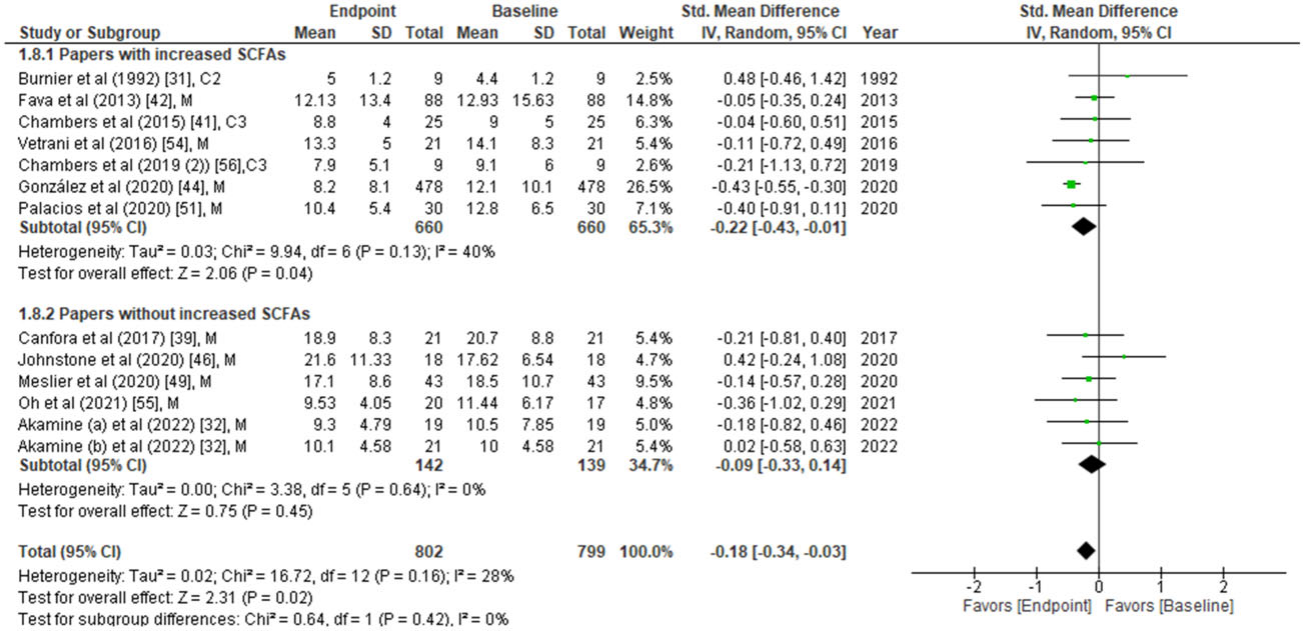
**Fasting insulin concentrations at baseline and endpoint (end of intervention) from each study in the treatment group**. Data are presented as the standardized mean difference (SMD) in fasting insulin (µU/mL) and have been separated into two subgroups: one with evidence of an increase in short-chain fatty acids (SCFAs) concentration(s) post intervention, and the other without any change in SCFAs post intervention. The type of intervention (M, meal/mixed; C3, propionate; C2, acetate) is noted at the end of each study. *Abbreviation:* IV, inverse variance.

**Figure 6:**
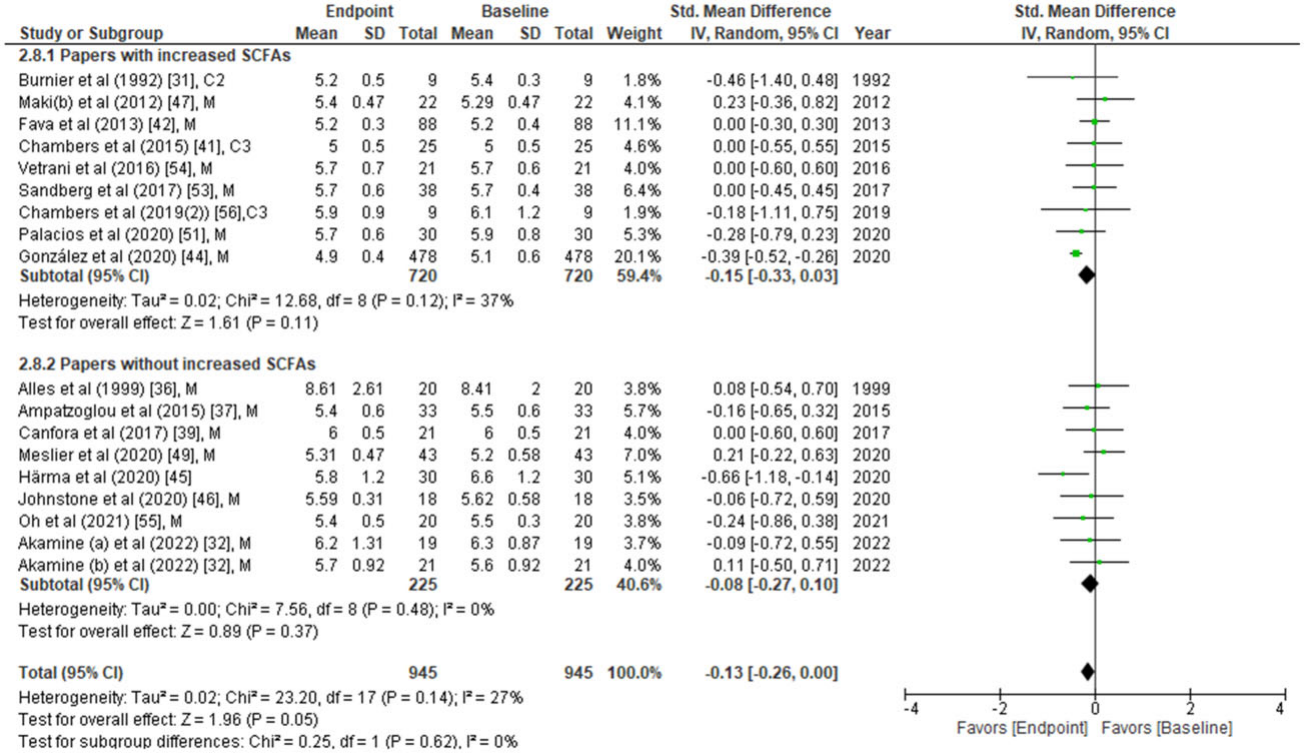
**Fasting glucose concentrations at baseline and endpoint (end of intervention) from each study in the treatment group**. Data are presented as the standardized mean difference (SMD) in fasting insulin (mmol/L) and have been separated into two subgroups: one with evidence of an increase in short-chain fatty acids (SCFAs) concentration(s) post intervention, and the other without any change in SCFAs post intervention. The type of intervention (M, meal/mixed; C3, propionate; C2, acetate) is noted at the end of each study. *Abbreviation*: IV, inverse variance.

**Figure 7:**
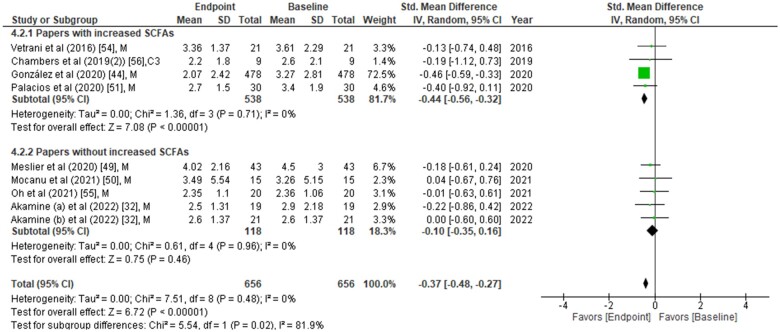
**Homeostatic model assessment of insulin resistance (HOMA-IR) values at baseline and endpoint (end of intervention) from available studies in the treatment group**. Data are presented as the standardized mean difference (SMD) in HOMA-IR and have been separated into two subgroups: one with evidence of an increase in short-chain fatty acids (SCFAs) concentration(s) post intervention, and the other without any change in SCFAs post intervention. The type of intervention (M, meal/mixed; C3, propionate) is noted at the end of each study. *Abbreviation*: IV, inverse variance.

Changes from baseline in fasting insulin, fasting glucose, and HOMA-IR were analyzed in the intervention group and the placebo group. A significantly lower fasting insulin level was observed at the end of intervention (Figure 5^31^^,^^32^^,^^39^^,^^41^^,^^42^^,^^44^^,^^46^^,^^49^^,^^51^^,^^54–56^) compared with baseline (SMD = −0.22; 95%CI = −0.43 to−0.01; *P* = 0.04) in studies that found increased SCFA concentrations. The overall effect on fasting insulin was also significant when all studies were included (SMD = −0.18; 95%CI = −0.34 to −0.03; *P* = 0.02; Figure 5). Fasting glucose did not demonstrate any significant changes relative to baseline in the meta-analysis (Figure 6^31^^,^^32^^,^^36^^,^^37^^,^^39^^,^^41^^,^^42^^,^^44–47^^,^^49^^,^^51^^,^^53–56^). On the other hand, HOMA-IR was significantly different (SMD = −0.44; 95%CI = −0.56 to −0.32; *P* < 0.00001) in studies with increased SCFA concentrations post intervention. This significance was also observed when all the studies (demonstrating increased or unchanged or decreased SCFAs post intervention) were included in the analysis (SMD = −0.37; 95%CI = −0.48 to −0.27; *P* < 0.00001) (Figure 7^32^^,^^44^^,^^49–51^^,^^54–56^). In the analysis of HOMA-IR, one study had more participants than others.^44^ The comparison of baseline vs endpoint values in the placebo group alone did not demonstrate any significant improvement in fasting insulin (see Figure S5 in the Supporting Information online),^39^^,^^41^^,^^46^^,^^49^^,^^51^^,^^54–56^ fasting glucose (see Figure S6 in the Supporting Information online),^36^^,^^37^^,^^39^^,^^41^^,^^46^^,^^47^^,^^49^^,^^51^^,^^53–56^ or HOMA-IR (see Figure S7 in the Supporting Information online).^49–51^^,^^54–56^

### Risk-of-bias assessment

The quality assessment of the 23 studies included in the meta-analysis is presented in Figure S8 in the Supporting Information online. As noted in the Methods section, crossover studies were analyzed separately (see Figure S8-A in the Supporting Information online).^36–38^^,^^40^^,^^43^^,^^46–48^^,^^52^^,^^53^ Several of the studies did not report sufficient details of unbiased data presentation or carryover effects to assign low or high risks, and thus they had to be classified as having unclear risk of bias. Eight studies did not describe allocation concealment, the criterion with the highest factor of unclear risk. Similarly, most studies did not have clear reporting of unbiased data (availability of study results at every time point during crossover). The risk of bias for blinding criteria was high in 6 studies. Since most of the studies were dietary interventions, blinding may not have been always applicable (eg, when the participants and the researchers could see which foods were given). In the parallel-arm trials, the risk of bias was much lower in most of the studies (see Figure S8-B in the Supporting Information online). Five treatment-only studies were not included in the analysis of selection, performance, or detection bias because all participants received the same treatment, and any selection/blinding was not applicable. These studies are represented by the white area in the graph (see Figure S8-B in the Supporting Information online).

## DISCUSSION

This systematic review and meta-analysis of 23 studies included a varied group of participants. The main finding was significantly lower fasting insulin in the group that received SCFAs directly and in the group that had evidence of an increase in SCFAs following intervention compared with the placebo group. When baseline and endpoint values were compared in the treatment groups, fasting insulin and HOMA-IR were significantly improved at the endpoint, a finding not observed for the baseline vs endpoint comparison in the placebo groups. Similar results were achieved when using the fixed-effects model, thus confirming the robustness of this study.

The underlying mechanism linking SCFAs to T2DM has not been fully elucidated. A mechanism involving gut microbes or microbial metabolites (eg, colonic SCFAs) regulating incretin hormone expression, insulin secretion, and glucose homeostasis is likely and needs validation. Incretin hormones such as glucose-dependent insulinotropic polypeptide and GLP-1 are known to regulate insulin secretion following oral glucose intake.^57^ Glucagon-like peptide 1 also plays a significant role in improving glucose tolerance and insulin sensitivity.^15^^,^^16^ Interestingly, SCFAs lowered fasting insulin and improved HOMA-IR without affecting fasting glucose. It would have been informative to understand the effects of the increase in SCFAs on GLP-1 concentrations, but only 6 studies measured GLP-1,^38–40^^,^^49^^,^^52^^,^^53^ and only 3 of these studies mentioned the use of the necessary dipeptidyl peptidase 4 inhibitors during the collection of samples.^39^^,^^52^^,^^53^ Meta-analysis to understand the effect of changes in SCFAs on GLP-1 was inconclusive in this same set of papers.

Sodium acetate, administered via gastric or intravenous infusion at various dosages, was the first SCFA to be investigated in human studies in the 1980s.^58–61^ Notably, most of the studies conducted during the last decade have focused on the oral administration of sodium propionate, sodium butyrate, or SCFA mixtures for extended periods of time (up to 6 months), with dosages ranging from 1 to 10 g/d. These studies and the long periods of SCFA intervention underscore the safety of SCFAs in human clinical studies/trials. To date, there have been no reports of any adverse effects of SCFAs on human health. A very recent systematic review and meta-analysis assessed the effects of acute and chronic administration of SCFAs and vinegar on glycemic control.^62^ Interestingly, the authors did not observe a significant effect of acute acetate or propionate treatment or chronic propionate treatment on glucose and insulin concentrations. However, they did not stratify their analyses on the basis of changes in SCFA concentrations post intervention. The quantitative analysis showed that only those studies with evidence of an increase in SCFA concentrations reported significantly lower fasting insulin values. Studies without any increase in SCFAs reported no change in insulin concentrations.

Short-chain fatty acids are typically measured in fecal or plasma samples. They are present in larger quantities in feces,^63^ and fecal SCFAs are considered to be an indirect representation of SCFA production in the colon. Short-chain fatty acids in the circulation are present in smaller quantities and usually reflect the amount remaining after uptake into colonocytes and then the liver.^64^ Nonetheless, circulating SCFAs are shown to be more directly associated with metabolic health than fecal SCFAs.^65^ The subanalysis also confirmed a significant effect of SCFAs on fasting insulin concentrations when circulating SCFAs are increased. One potential reason for higher variability in fecal SCFAs could be the variations in methods used for collection, storage, and processing of samples.^66^ Standardization of methods for measurement of SCFAs is urgently needed.

The present meta-analysis demonstrates that direct administration of SCFAs is more beneficial than indirect methods of administration, including a variety of diets. It is notable that SCFAs are not well suited to oral administration because of their unpleasant taste and odor.^67^ Recent studies have focused on the development of novel targeted delivery systems that can deliver SCFAs into the colon or the circulatory system.^68^ Only one study in the present systematic review investigated the direct infusion of SCFAs (ie, acetate), reporting increases in circulating (plasma/serum) SCFAs.^31^ All the remaining studies that provided SCFAs as capsules, powders, or meals reported variable SCFA concentrations at the endpoint.^38^^,^^40^^,^^41^^,^^56^

Apart from potential differences in fecal and circulating SCFA concentrations, another reason for this variation could be different methods of assessing compliance. Three trials maintained regular communication between participants and investigators to encourage high compliance, and the participants returned their used and unused sachets to calculate the compliance rate at the end of the study.^40^^,^^41^^,^^56^ Future studies should ensure that compliance with the intervention is assessed and reported.

Because of the invasive nature of infusion studies, the direct infusion of acetate was performed for only a short period of time.^31^ However, studies that confirmed an increase in SCFA concentrations (Figure 2) were those that reported a lowering effect on fasting insulin.^40^^,^^41^^,^^47^^,^^48^^,^^51–54^^,^^56^ Therefore, it would be interesting to understand the long-term effects of SCFA infusion on insulin sensitivity. Clinical trials addressing this question are warranted. Interestingly, a separate systematic review and meta-analysis confirmed that the left colon may contribute to the maintenance of glucose homeostasis.^69^ Since human GLP-1-producing L cells are found at the highest density in the left colon,^70^ removal of the left colon is, not surprisingly, associated with T2DM. The direct infusion of SCFAs provides a unique opportunity to manipulate the expression of incretin hormones, and more studies in this area are needed.

Dietary fiber (from whole grain, oat/wheat/barley bran, guar gum, pectin, legumes, psyllium, and resistant starch) enhances the production of SCFAs in the colon following fermentation by gut microbes.^71^^,^^72^ Although the beneficial effects of dietary fiber intake on obesity and diabetes have been highlighted,^73^^,^^74^ this meta-analysis provides evidence for the beneficial role of SCFA interventions in lowering fasting hyperinsulinemia (see Figures S1 and S2 in the Supporting Information online). Short-chain fatty acids are naturally available through fermented beverages and food products, such as apple cider vinegar, kombucha, fermented alcoholic beverages, kefir, cheese, legumes, nuts/seeds, soybean paste, ghee, sauerkraut, and pickled vegetables.^75^^,^^76^ These food products contain high levels of SCFAs, are easy to obtain, and provide readily available dietary sources of SCFAs. Daily SCFA consumption (in moderation) through SCFA-rich food products may hold great potential to improve insulin sensitivity and provide substantial health benefits, including reducing the risk of cancer.^77^ Larger clinical studies investigating the effects of these fermented beverages and food products are warranted.

The limitations of this meta-analysis are mainly due to the heterogeneity of the included studies, which was unavoidable because of the nature of the study question around the effect of SCFAs on insulin sensitivity. The factors contributing to the high heterogeneity were age, sex, sample size, diet, lifestyle, underlying health conditions, types of SCFAs measured (acetate, propionate, butyrate, or all), and the source of SCFAs (feces or plasma). Other sources of heterogeneity include the different types of interventions used, such as various diets, gastric bypass surgery, or SCFAs provided as capsules or via infusion. It was not possible to perform subgroup analyses to understand the effects of SCFA dosage or SCFA delivery route on fasting insulin and fasting glucose because the number of studies was too low. It is also notable that majority of the studies in this analysis were conducted on 3 different continents (Europe, North America, and Australia), which may have contributed to the risk of bias through the recruitment of the majority of individuals of only one ethnicity. However, the ethnicity of participants was not mentioned in any of the studies. The included studies reported differences in sensitivities of the methods used for measurement of SCFAs and insulin or glucose. In addition, the literature search was limited to papers published in English, and thus 169 non-English papers were excluded.

Despite its limitations, this systematic review is the first to summarize and provide a meta-analysis of clinical studies showing the beneficial effects of increased SCFAs on fasting insulin and HOMA-IR. This meta-analysis captured all studies that reported SCFA changes and provided actual values of fasting insulin and glucose in humans. This approach was chosen as the focus of the systematic review and meta-analysis in order to increase current understanding of the metabolic benefits of elevated SCFAs following clinical interventions.

## CONCLUSION

This is the first meta-analysis to demonstrate a significant decrease in fasting insulin when SCFA concentrations were confirmed to have increased. While this beneficial effect of SCFAs on fasting insulin has been confirmed, several factors need to be evaluated before optimal implementation of SCFA interventions, such as the type of SCFA (acetate, propionate, butyrate, or a mixture), the form of SCFA administration (fiber-enriched diets or capsules containing sodium salts or inulin esters), the duration of intervention, the route of delivery (oral or intravenous infusion), and patient compliance. Nonetheless, on the basis of the overall results presented here, direct SCFA consumption or an SCFA-enriched diet was found to have beneficial effects on fasting insulin concentrations. Therefore, SCFA interventions, or dietary guidance along with clinical monitoring and lifestyle management, could be considered a safe and novel treatment for individuals with overweight, obesity, or T2DM.

## Supplementary Material

nuad042_Supplementary_Data

## Supporting Information

The following Supporting Information is available through the online version of this article at the publisher’s website.

*Appendix S1* **PRISMA 2020 checklist.**

*Table S1* **Previously published relevant meta-analyses and systematic reviews assessing the effects of diet, pre-/pro-/synbiotics on insulin and glucose metabolism.**

*Table S2* **Concentrations of short-chain fatty acids (SCFAs) reported in different studies for placebo and treatment groups at the end of intervention.**

*Figure S1* **Forest plot of fasting insulin concentrations between control group and treatment subgroup by direct or indirect administration of short-chain fatty acids.**

*Figure S2* **Forest plot of fasting insulin concentrations between control group and treatment subgroup by direct or indirect administration of short-chain fatty acids (SCFAs) and by increase or no increase in SCFAs.**

*Figure S3* **Forest plot of fasting insulin concentrations between control group and treatment subgroup by measurement of plasma or fecal short-chain fatty acids (SCFAs) and by increase or no increase in SCFAs.**

*Figure S4* **Correlation matrix between changes in concentrations of fasting insulin, fasting glucose, and different short-chain fatty acids.**

*Figure S5* **Forest plot of fasting insulin concentrations between baseline and endpoint in placebo group.**

*Figure S6* **Forest plot of fasting glucose concentrations between baseline and endpoint in placebo group.**

*Figure S7* **Forest plot of homeostatic model assessment of insulin resistance (HOMA-IR) between baseline and endpoint in placebo group.**

*Figure S8* **Risk-of-bias analysis.**
